# Fluctuations in Serum Creatinine Levels During Hospitalization and Long-Term End-Stage Kidney Disease and Mortality

**DOI:** 10.1001/jamanetworkopen.2023.26996

**Published:** 2023-08-03

**Authors:** Orly Efros, Pazit Beckerman, Ayelet A. Basson, Roy Cohen, Eyal Klang, Yael Frenkel Nir, Shelly Soffer, Noam Barda, Ehud Grossman

**Affiliations:** 1 National Hemophilia Center and Thrombosis & Hemostasis Institute, Sheba Medical Center, Ramat Gan, Israel; 2 Sackler Faculty of Medicine, Tel Aviv University, Tel Aviv, Israel; 3 Institute of Nephrology and Hypertension, Sheba Medical Center, Ramat Gan, Israel; 4 TIMNA–Israel National Big Data Platform for Health Research, Ministry of Health, Jerusalem, Israel; 5 ARC Innovation Center, Sheba Medical Center, Ramat Gan, Israel; 6 Department of Medical Management, Sheba Medical Center, Ramat Gan, Israel; 7 Internal Medicine B, Assuta Medical Center, Ashdod, Israel; 8 Ben-Gurion University of the Negev, Be’er Sheva, Israel; 9 Software and Information Systems Engineering, Ben Gurion University, Be’er Sheva, Israel; 10 Epidemiology, Biostatistics, and Community Health Services, Ben-Gurion University of the Negev, Be’er Sheva, Israel; 11 Internal Medicine Wing, Sheba Medical Center, Ramat Gan, Israel

## Abstract

**Importance:**

Acute kidney injury is associated with poor outcomes, but the clinical implication of reversible serum creatinine level fluctuations during hospitalization not necessarily defined as acute kidney injury is poorly understood.

**Objective:**

To investigate the long-term outcomes of patients without previously diagnosed kidney disease who present with decreased kidney function and are subsequently discharged with apparently normal kidney function.

**Design, Setting, and Participants:**

A retrospective cohort study was conducted of patients hospitalized in a large tertiary hospital in Israel between September 1, 2007, and July 31, 2022. The study included patients admitted to an internal medicine ward. Patients had not undergone dialysis during the index hospitalization, had at least 3 creatinine tests performed during hospitalization, and had a discharge estimated glomerular filtration rate (eGFR) exceeding 60 mL/min/1.73 m^2^. Patients with preexisting chronic kidney disease were excluded.

**Exposure:**

Glomerular filtration rate was estimated from serum creatinine values using the updated 2022 Chronic Kidney Disease Epidemiology Collaboration formula, and eGFR greater than 60 mL/min/1.73 m^2^ was regarded as normal. Exposure was defined based on the association between the first and last values determined during hospitalization.

**Main Outcomes and Measures:**

All-cause mortality in the year following the index hospitalization and end-stage kidney disease (ESKD) in the 10 years following the index hospitalization.

**Results:**

A total of 40 558 patients were included. Median age was 69 (IQR, 56-80) years, with 18 004 women (44%) and 22 554 men (56%). A total of 34 332 patients (85%) were admitted with a normal eGFR and 6226 (15%) with decreased eGFR. Patients with decreased eGFR on presentation had an 18% increased mortality in the year following hospitalization (adjusted hazard ratio [AHR], 1.18; 95% CI, 1.11-1.24) and a 267% increased risk of ESKD in the 10 years following hospitalization (AHR, 3.67; 95% CI, 2.43-5.54), despite having been discharged with apparently normal kidney function. The highest risk was noted in patients who presented to the hospital with an eGFR of 0 to 45 mL/min/1.73 m^2^.

**Conclusions and Relevance:**

The findings of this cohort study suggest that patients who present with decreased kidney function and are discharged without clinically evident residual kidney disease may be at increased long-term risk for ESKD and mortality.

## Key Points

**Question:**

What are the long-term outcomes for hospitalized patients with reduced kidney function on admission who are discharged with apparently normal kidney function?

**Findings:**

In this cohort study that included 40 558 adults, patients with reduced kidney function on admission who were discharged with apparently normal kidney function experienced 18% increased mortality in the year following the hospitalization. The risk of end-stage kidney disease increased by 267% in the 10 years following the hospitalization.

**Meaning:**

The findings of this study suggest that reversible reduction in kidney function among hospitalized patients is associated with an increased long-term risk for end-stage kidney disease and mortality.

## Teaser

This cohort study evaluates 1- and 10-year mortality of patients with decreased estimated glomerular filtration rate on admission whose kidney function appeared to have returned to normal at discharge.

## Introduction

The global incidence of acute kidney injury (AKI) is rapidly increasing, particularly among hospitalized patients with acute illness.^1,2^ In a systematic review of large cohort studies, which included 49 million patients, 1 in 5 adults worldwide experienced AKI during hospitalization.^3^

Several studies have reported that AKI during hospitalization is associated with poor outcomes and high use of health care resources.^1,4,5,6,7,8,9^ A meta-analysis of 49 studies containing a total of 47 017 participants noted that patients who developed AKI during hospitalization had a higher rate of long-term mortality than patients without AKI during hospitalization.^7^ In a large observational study of 104 764 hospitalized veterans without chronic kidney disease (CKD), even a mild episode of in-hospital AKI with full recovery was associated with the eventual development of CKD.^10^

The Kidney Disease Improving Global Outcomes (KDIGO) staging system defines AKI as an increase in the serum creatinine level over hours to days (ie, ≥0.3 mg/dL within 48 hours or ≥1.5 times baseline within the previous 7 days [to convert to micromoles per liter, multiply by 88.4]) or a decrease in urine output (≤0.5 mL/kg/h for 6 hours).^11^ Therefore, AKI may only partially represent the prognostic importance of serum creatinine level fluctuations during hospitalization.

Indeed, worsening kidney function was also noted to be a predictor of increased mortality, even when not meeting the definition of AKI.^12,13,14,15^ Givertz et al^14^ examined data from 1962 patients with acute heart failure and kidney dysfunction from the PROTECT study. In this analysis, an increase of more than 0.1 mg/dL per day in serum creatinine levels increased mortality risk, whereas stable or decreasing creatinine levels were associated with reduced risk.

However, the clinical implication of creatinine level changes, which are not necessarily defined as AKI, with subsequent full recovery, is poorly described. This study aimed to investigate the long-term outcome of patients without previously diagnosed kidney disease who presented with decreased kidney function but may not have met the full AKI criteria^16^ and were subsequently discharged from the hospital with apparently normal kidney function.

## Methods

### Study Setting and Design

This was a retrospective cohort study based on the integration of the electronic medical records from Sheba Medical Center (SMC) with Israeli nationwide records regarding end-stage kidney disease (ESKD) using TIMNA, a national research platform established by the Israeli government for conducting big-data research and combining deidentified health data from multiple health organizations. All medical data generated by SMC are recorded in electronic medical records and subsequently stored in an analytic data warehouse for further study. By a directive in the national health care insurance law, data regarding all patients in Israel with ESKD (chronic dialysis treatment and kidney transplant) are collected in a central registry maintained by the Ministry of Health. These data are manually verified and constantly updated. The mortality status of patients was identified using the official death records maintained by the Ministry of Interior in the State of Israel, which provides comprehensive and up-to-date information on all deaths occurring within the country. The study was approved by the SMC Institutional Review Board and was exempt from the requirement for informed consent due to the use of deidentified data. The reporting of this study aligns with the Strengthening the Reporting of Observational Studies in Epidemiology (STROBE) reporting guideline. Patients included in the study were those admitted to an internal medicine ward in SMC between September 1, 2007, and July 31, 2022, were older than 18 years at hospitalization, were hospitalized between 2 and 14 days, had not undergone dialysis during the index hospitalization, and had at least 3 creatinine tests performed during their hospitalization. Although our primary analysis focused on the first and last creatinine values, by mandating 3 or more creatinine level determinations, we indirectly ensured sufficient monitoring of kidney function during the patients’ hospitalizations. For patients admitted multiple times during the study period, only the first hospitalization was retained.

Glomerular filtration rate (GFR) was estimated from creatinine values using the Chronic Kidney Disease Epidemiology Collaboration formula, updated for the year 2022.^17^ Only patients with discharge estimated GFR (eGFR) values over 60 mL/min/1.73 m^2^ were included.

Individuals with a history of CKD, dialysis therapy, or kidney transplant, as noted by the admitting physician, were excluded from the study. Documentation of CKD was identified in the admission file by the relevant *International Statistical Classification of Diseases and Related Health Problems: Alphabetical Index* codes^18^ and diagnosis-free text (eTable 1 in Supplement 1 provides the full code listing).

Exposure was defined based on the association between the first and last eGFR measurements performed during hospitalization. Patients for whom both the first and last values were over 60 mL/min/1.73 m^2^ were defined as having normal to normal kidney function. Patients for whom the first value was under 60 mL/min/1.73 m^2^ and the last value was over 60 mL/min/1.73 m^2^ were defined as having low to normal kidney function. To account for minor test-to-test variations and ensure we were capturing a substantial change in kidney function, patients were included in the low-to-normal kidney function category only if the increase in eGFR was 15% or more of the initial value.

Two outcomes were considered. The first was all-cause mortality in the year following the index hospitalization. The second was ESKD in the 10 years following the index hospitalization. Covariates for adjustment were the same for both outcomes, were chosen based on domain expertise, and included age, sex, and a history of hypertension, diabetes, ischemic heart disease, heart failure, cancer, atrial fibrillation, and chronic obstructive pulmonary disease. Additional variables were included to describe the study population. The specific criteria used to identify each condition are detailed in eTable 1 in Supplement 1.

Several additional analyses were performed. First, subgroup analyses were performed for groups defined by age, sex, background diseases, and treatment with either angiotensin-converting enzyme inhibitors or angiotensin II receptor blockers. We then considered the exposure in a more refined manner, separating patients with a first eGFR value between 45 and 60 mL/min/1.73 m^2^ and patients with a first eGFR value of less than 45 mL/min/1.73 m^2^. We further adjusted for discharge eGFR to directly compare patients with the same discharge kidney function. We also performed the main analysis allowing a different baseline hazard across age groups. For a sensitivity analysis, we changed the normal eGFR threshold to 90 mL/min/1.73 m^2^.

To ensure that normal eGFR on discharge was not due to decreased creatinine levels secondary to factors other than improved kidney function, such as loss of muscle mass during critical illness,^19^ we conducted numerous analyses to address further possible misclassification, including a subgroup analysis of patients with a hospital stay of 5 days or less and those hospitalized for more than 5 days and a subgroup analysis that only included patients in the low-to-normal kidney function category if they had an increase in eGFR of 30% or more.

We explored temporal trends in mortality and ESKD over the study period by estimating the interaction between primary exposure and calendar time. Finally, we also defined the exposure solely based on creatinine levels, without relying on eGFR. For this analysis, we considered 2 groups: patients with admission creatinine levels above the normal limit (1.3 mg/dL for men and 1.1 mg/dL for women) and patients with admission creatinine levels below the normal limit, both with normal creatinine levels on discharge. To ensure sufficient kidney function changes, we still required at least a 15% improvement in creatinine levels for the low-to-normal group.

### Statistical Analysis

The study population was described in terms of sociodemographic variables, physical measurements, and background medical conditions. Appropriate statistical tests were selected for each variable. Crude survival curves for each outcome were calculated using the Kaplan-Meier estimator, stratified by the exposure, and compared using the log-rank test.

Cox proportional hazards regression was used to estimate the hazard ratio (HR) for each outcome between the 2 exposure groups over the study period, adjusted for the aforementioned variables. The assumption of proportionality of hazards was checked using Schoenfeld residuals.

Missing data, which are rare in the SMC database for the variables used in this study, were handled using a complete case analysis. The analysis was performed using the R statistical software, version 4.1.2 (R Foundation for Statistical Computing). The significance threshold, set at *P* < .05, was unpaired and 2-sided.

## Results

Following the application of the eligibility criteria, 40 558 individuals were included in the study; of these, 34 332 (85%) belonged to the normal-to-normal group and 6226 (15%) to the low-to-normal group. A total of 765 patients were excluded due to not meeting the 15% or more increase in eGFR from the initial value required for inclusion in the low-to-normal category. The median age was 69 (IQR, 56-80) years, with 18 004 women (44%) and 22 554 men (56%). Patients in the low-to-normal group were older; had a higher prevalence of comorbid conditions, such as diabetes, ischemic heart disease, heart failure, chronic obstructive pulmonary disease, hypertension, and atrial fibrillation; and were more likely to be treated with diuretics or renin-angiotensin-aldosterone system inhibitors on admission and discharge (Table 1; eTable 2 in Supplement 1). A total of 1195 patients in the low-to-normal group had a further decrease in eGFR during hospitalization before the eGFR increased to the normal range.

**Table 1.** Demographic and Clinical Characteristics of the Study Population at Baseline

Variable	Total, No. (%) (N = 40 558)	Kidney function, No. (%)		*P* value
		Normal to normal (n = 34 332)	Low to normal (n = 6226)	
Age, median (IQR), y	69 (56-80)	67 (54-78)	78 (69-85)	<.001
Sex				
Women	18 004 (44)	14 924 (43)	3080 (49)	<.001
Men	22 554 (56)	19 408 (57)	3146 (51)	
Hemoglobin, median (IQR), g/dL^a^	12.4 (10.9-13.8)	12.5 (11.0-13.8)	11.9 (10.5-13.3)	<.001
First creatinine, median (IQR), mg/dL	0.89 (0.72-1.08)	0.83 (0.70-0.98)	1.34 (1.14-1.58)	<.001
Last creatinine, median (IQR), mg/dL	0.81 (0.68-0.96)	0.80 (0.66-0.93)	0.90 (0.80-1.06)	<.001
Systolic blood pressure, median (IQR), mm Hg^b^	125 (110-142)	125 (110-142)	123 (108-141)	<.001
Ischemic heart disease	8626 (21)	7363 (21)	1263 (20)	.04
Heart failure	2812 (7)	2083 (6)	729 (12)	<.001
Cancer	7583 (19)	6393 (19)	1190 (19)	.40
Chronic obstructive pulmonary disease	2522 (6)	2049 (6)	473 (8)	<.001
Atrial fibrillation	5078 (13)	3935 (11)	1157 (19)	<.001
Hypertension	18 720 (46)	15 161 (44)	3559 (57)	<.001
Cerebrovascular accident	3475 (9)	2676 (8)	799 (13)	<.001
Diabetes	10 312 (25)	8387 (24)	1925 (31)	<.001
Furosemide	4589 (11)	3277 (10)	1312 (21)	<.001
Thiazide	1548 (4)	1203 (4)	345 (6)	<.001
Spironolactone	1720 (4)	1295 (4)	425 (7)	<.001
Angiotensin-converting enzyme inhibitor	8599 (21)	6808 (20)	1791 (29)	<.001
Angiotensin II receptor blocker	5561 (14)	4445 (13)	1116 (18)	<.001
Length of stay, median (IQR), d	4 (3-6)	4 (3-6)	5 (3-7)	<.001

SI conversion: To convert creatinine to micromoles per liter, multiply by 88.4; hemoglobin to grams per liter, multiply by 10.

^a^ Data missing for 0.64% of the population.

^b^ Data missing for 0.39% of the population.

**Figure 1.**
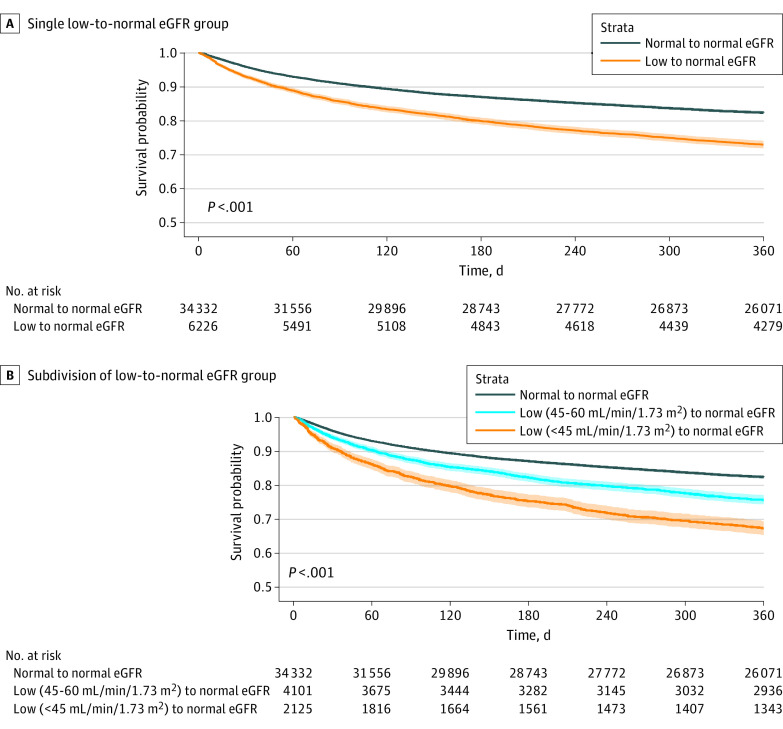
One-Year Survival Following Hospitalization Estimates for survival in the year following hospitalization, with a single low-to-normal estimated glomerular filtration rate (eGFR) exposure group (A) and with a subdivision of the low-to-normal eGFR exposure group (B). Patients for whom both the first and last eGFR values were over 60 mL/min/1.73 m^2^ were defined as having normal to normal kidney function. Patients for whom the first value (ie, at presentation) was under 60 mL/min/1.73 m^2^ and the last value (ie, at discharge) was over 60 mL/min were defined as having low to normal kidney function. The low to normal group was subdivided into 2 levels: less than 45 mL/min/1.73 m^2^ at presentation and 45 to 60 mL/min/1.73 m^2^ at presentation. Shaded areas indicate 95% CI.

**Figure 2.**
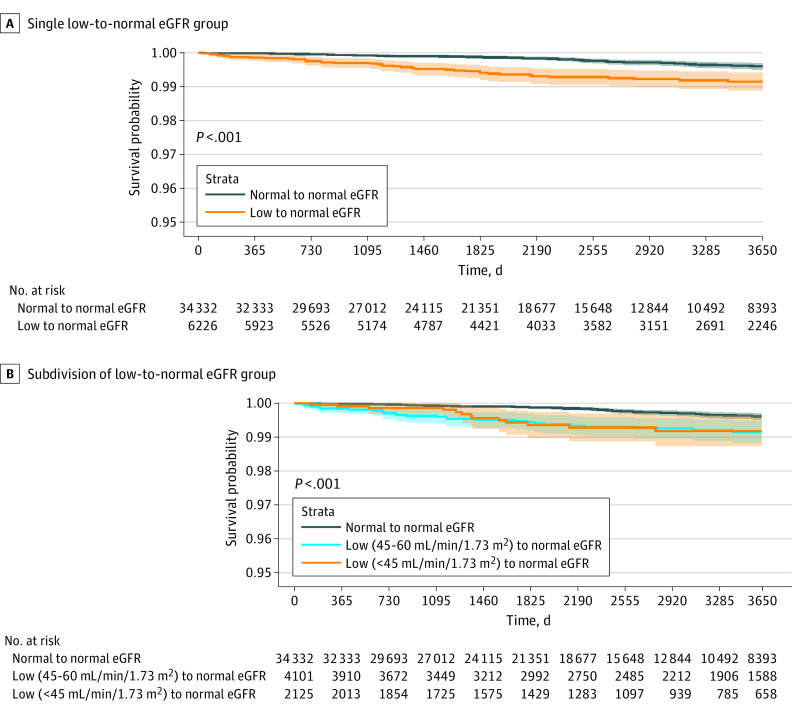
Ten-Year Probability for End-Stage Kidney Disease Estimated glomerular filtration rate (eGFR) for end-stage kidney disease in the 10 years following hospitalization, with a single low-to-normal eGFR exposure group (A) and a subdivision of the low-to-normal eGFR exposure group (B). Shaded areas indicate 95% CI.

Crude analysis showed increased mortality in the year following the index hospitalization (Figure 1A) and increased risk for ESKD in the 10 years following the index hospitalization (Figure 2) in the low-to-normal group. The adjusted analysis estimated an 18% increased risk of death for the low-to-normal group in the year following hospitalization compared with the normal-to-normal group (adjusted HR [AHR], 1.18; 95% CI, 1.11-1.24) and a 267% increased risk for ESKD for the low-to-normal group in the 10 years following hospitalization compared with the normal-to-normal group (AHR, 3.67; 95% CI, 2.43-5.54). No substantial heterogeneity of the association was observed when looking at different age groups, sex, or specific comorbidities (Table 2; eTable 4 in Supplement 1). Inspecting Schoenfeld residuals, we found that the main exposure obeyed the proportional hazards assumption (eFigure in Supplement 1).

**Table 2.** The Association Between Kidney Function at Admission and Outcomes Following the Index Hospitalization^a^

Population	Adjusted hazard ratio (95% CI)	
	Mortality	End-stage kidney disease
Entire study cohort	1.18 (1.11-1.24)	3.67 (2.43-5.54)
Granular exposure groups		
Admission eGFR 45-60 mL/min/1.73 m^2^	1.05 (0.98-1.13)	3.76 (2.37-5.96)
Admission eGFR <45 mL/min/1.73 m^2^	1.42 (1.31-1.54)	3.49 (1.90-6.43)
Age ≥70 y	1.18 (1.10-1.25)	2.91 (1.31-6.47)
Age <70 y	1.13 (0.99-1.27)	4.30 (2.73-6.78)
Men	1.17 (1.08-1.27)	3.40 (2.06-5.60)
Women	1.18 (1.09-1.28)	4.35 (2.11-8.98)
Diabetes	1.13 (1.01-1.25)	4.04 (2.40-6.79)
Hypertension	1.19 (1.10-1.28)	3.52 (2.09-5.93)
ACEI or ARB treatment	1.27 (1.16-1.38)	3.39 (2.01-5.74)

Abbreviations: ACEI, angiotensin-converting enzyme inhibitor; ARB, angiotensin II receptor blocker; eGFR, estimated glomerular filtration rate.

^a^ Adjusted analysis of the association between these low-to-normal eGFR groups and outcomes compared with the normal-to-normal group: mortality in the year following the index hospitalization and end-stage kidney disease in the 10 years following the index hospitalization. The analysis was performed using a Cox proportional hazards model, adjusted for age, sex, and a history of hypertension, ischemic heart disease, heart failure, cancer, and chronic obstructive pulmonary disease. When a variable was used to define a subgroup, it was not used for adjustment (eg, the analysis within the subgroup with diabetes was not adjusted for diabetes).

For the mortality outcome in the full study population and within subgroups a dose-response association was evident, with worse outcomes for patients whose admission eGFR was lower (Figure 1B, Table 2). In the full population, we estimated a higher risk of death in patients with an eGFR of 0 to 45 mL/min/1.73 m^2^ on admission (AHR, 1.42; 95% CI, 1.31-1.54) than for patients with an eGFR of 45 to 60 mL/min/1.73 m^2^ (AHR, 1.05; 95% CI, 0.98-1.13).

Further adjustment for discharge eGFR did not substantially alter the estimates (eTable 3 in Supplement 1), nor did allowing a different baseline hazard by age group (eTable 4 in Supplement 1). In our sensitivity analysis using an eGFR threshold of 90 mL/min/1.73 m^2^, we did not find an association between the exposure group and 1-year mortality (AHR, 0.95; 95% CI, 0.87-1.04) and did not have enough data to analyze the risk for 10-year ESKD.

Subgroup analyses of patients with a hospital stay of 5 days or less, those hospitalized for more than 5 days, and a subgroup from the low-to-normal group with an increase in eGFR of 30% or more showed a persistently higher risk of 1-year mortality and ESKD within 10 years in the low-to-normal group compared with the normal-to-normal group (eTable 4 in Supplement 1). Interaction analysis did not reveal a significant temporal trend in the incidence of 1-year-mortality (interaction HR, 1.00; 95% CI, 1.00-1.03) or 10-year risk for ESKD (interaction HR, 1.01; 95% CI, 0.89-1.14) over the study timeframe.

The results from our creatinine level–based analysis mirrored the trends we observed in our eGFR-based analysis. The adjusted analysis estimated a 25% increased risk of death in the year following hospitalization for patients with admission creatinine levels above the normal limit that subsequently decreased upon discharge compared with patients with admission and discharge normal creatinine levels (AHR, 1.25; 95% CI, 1.18-1.33). A 209% increased risk for ESKD was found for patients with admission creatinine levels above the normal limit that subsequently decreased upon discharge compared with patients with admission and discharge normal creatinine levels (AHR, 3.09; 95% CI, 2.02-4.71).

## Discussion

This study of a large cohort of hospitalized patients discharged with an apparently normal eGFR, defined as eGFR greater than or equal to 60 mL/min/1.73 m^2^, noted a higher risk of mortality and ESKD among participants with decreased eGFR on admission that was not necessarily confined to the KDIGO definition of AKI^11^ compared with participants who presented with a normal eGFR. Our findings remained consistent when defining the exposure solely based on creatinine levels. The risk seemed to be graded, with the highest risk for mortality noted in patients who presented to the hospital with an eGFR of less than 45 mL/min/1.73 m^2^ than with an eGFR of 45 to 60 mL/min/1.73 m^2^. This increased risk persisted after adjusting for confounders; stratifying by age groups, sex, background disease, and treatment with either angiotensin-converting enzyme inhibitors or angiotensin II receptor blockers; and when allowing a different baseline HR across age groups. It was not altered by adjusting for discharge eGFR, and we did not observe temporal trends. These results suggest that patients who present with decreased kidney function and are discharged without clinically evident residual kidney disease are at increased long-term risk of ESKD.

The high 1-year mortality rate observed in our study reflects the patient cohort, which primarily consists of older individuals with acute or complex conditions. Similar trends have been noted in other studies, such as that by Fløjstrup et al,^16^ emphasizing the serious outcomes often associated with acute hospital admissions in cohorts with these demographic characteristics.

The association between recovered kidney injury and long-term clinical outcomes was previously described.^10,20,21,22^ Bucaloiu et al^20^ found an increased mortality risk and future CKD following an in-hospital reversible AKI among 1610 patients without preexisting kidney disease. Similar findings were found in other studies, with a consistent association between reversible AKI and subsequent clinical outcomes, including subsequent CKD and mortality rate.^22,23^ However, kidney outcomes and mortality risk following any reduction in GFR with subsequent full recovery were not studied. Moreover, current guidelines do not recommend long-term follow-up of patients with non-AKI.^11^ To our knowledge, this is the first study to investigate the mortality risk and ESKD among a large cohort of patients discharged from the hospital with apparently recovered kidney function following any eGFR reduction and without necessarily meeting time-dependent creatinine level–based KDIGO-defined AKI.

Creatinine level–based eGFR may not accurately reflect the severity of the kidney injury in the acute setting, as creatinine levels are often not increased until several days after AKI has occurred.^24^ Despite this shortcoming, the use of creatinine level–based eGFR has distinct advantages compared with current risk stratification strategies, which focus on the presence of AKI and an increase in serum creatinine levels over time.^7,11,25,26,27^ First, reliance on the definition of AKI may fail to recognize clinical outcomes of patients with smaller serum creatinine increases or variable creatinine level fluctuations. Second, these approaches still require information on baseline serum creatinine levels, which is not always available. In addition, efforts to estimate baseline creatinine levels from in-hospital creatinine tests or by using other patient-based equations were inaccurate and resulted in misclassifications of AKI.^28^

A possible explanation for our findings is that an observed decrease in kidney function during hospitalization might reveal an existing decreased kidney reserve, unmasked by the stress of acute illness.^29^ Even though kidney function may seem to be recovered at discharge, the persistent reduction in kidney reserve could have long-term implications. Setting the threshold for normal kidney function at an eGFR of 90 mL/min/1.73 m^2^ revealed no associations with 1-year mortality, implying that more substantial kidney damage may be required to influence long-term outcomes.

### Limitations

Our study has several limitations. We did not have access to the baseline prehospitalization serum creatinine levels of the patients included in our study and therefore cannot definitively establish a full recovery to baseline eGFR. We included only patients with 3 or more creatinine tests during hospitalization, which could result in selectively including patients admitted for more severe medical conditions requiring serial blood testing. We included a heterogeneous hospitalized population with different admission diagnoses and etiologies for reduced kidney function on presentation. We only considered a single hospitalization per patient, because evaluating further hospitalizations was beyond the scope of our study and limited by our lack of access to patient data from other medical institutions.

The main challenge to the robustness of our results could be the potential misclassification of exposure groups. Relying on creatinine levels to estimate GFR values may not perfectly represent the measured GFR on an individual level and could result in misclassification by several mechanisms.^24,30^ First, it is possible that creatinine levels have not yet reached their steady-state peak at the time of measurement, leading to some participants with low-to-normal kidney function being misclassified as having normal-to-normal kidney function. However, such misclassification would likely result in an underestimation of the long-term risks for ESKD and mortality in the low-to-normal group in our findings. Second, Prowle et al^19^ described a decrease in discharge serum creatinine levels among patients hospitalized in intensive care units, attributed by the authors to a decrease in muscle mass over time during critical illness. This could have potentially misclassified some patients with low to normal eGFR as having normal to normal eGFR. However, the patient population in our study had a substantially shorter hospital stay than those in the Prowle et al study, and a subgroup analysis of patients hospitalized for less than 5 days showed consistent results, limiting the potential for extensive muscle mass loss. The inclusion criteria for the low-to-normal group of at least a 15% eGFR improvement and the additional analysis of patients with more than 30% eGFR improvement indicated substantial creatinine level change unlikely due to critical illness alone. Here again, an underestimation of discharge creatinine levels due to factors other than recovery reinforces our conclusions about the need for long-term follow-up for these patients. In addition, our study could have encountered potential misclassification by including patients with CKD. However, CKD is conventionally indicated by an eGFR of less than 60 mL/min/1.73 m^2^ for a period exceeding 3 months and has implications for health, as per the KDIGO guidelines.^31^ By selectively including patients with restored normal eGFR at discharge and excluding those with a previous CKD diagnosis recorded by the admitting physician, this risk is mitigated.

## Conclusions

The findings of this cohort study suggest that, in hospitalized patients discharged with apparently recovered kidney function, a decreased eGFR on presentation may be associated with increased mortality in the year following the hospitalization and increased risk of ESKD in the 10 years following the hospitalization. Currently, these populations are not considered at risk unless AKI, as defined by KDIGO, was observed. Therefore, these patients should be advised to continue medical observation following their discharge from the hospital.

## Notes

Supplement 1. **eTable 1.** Definitions of Variables Used for the Exposure, Eligibility, and Adjustment **eTable 2.** Treatment Frequency of Diuretics or Renin-Angiotensin-Aldosterone System Inhibitors on Admission and Discharge **eTable 3.** The Association Between Kidney Function at Admission and Outcomes Following the Index Hospitalization With Adjustment to eGFR at Discharge **eTable 4.** Additional Analyses of the Association Between Kidney Function at Admission and Outcomes Following the Index Hospitalization **eFigure.** Proportionality of Hazards of the Main Exposure Assessed by Schoenfeld Residuals

Supplement 2.
Data Sharing Statement
