# Supplementary material for: Fluctuations in Serum Creatinine Levels During Hospitalization and Long-Term End-Stage Kidney Disease and Mortality
Source: JAMA Netw Open. 2023 Aug 3;6(8):e2326996. doi: 10.1001/jamanetworkopen.2023.26996 (PMC10401303; doi:10.1001/jamanetworkopen.2023.26996)
Supplement: Supplement 1. — eTable 1. Definitions of Variables Used for the Exposure, Eligibility, and Adjustment eTable 2. Treatment Frequency of Diuretics or Renin-Angiotensin-Aldosterone System Inhibitors on Admission and Discharge eTable 3. The Association Between Kidney Function at Admission and Outcomes Following the Index Hospitalization With Adjustment to eGFR at Discharge eTable 4. Additional Analyses of the Association Between Kidney Function at Admission and Outcomes Following the Index Hospitalization eFigure. Proportionality of Hazards of the Main Exposure Assessed by Schoenfeld Residuals [file jamanetwopen-e2326996-s001.pdf]

Supplementary Online Content

Efros O, Beckerman P, Basson AA, et al. Fluctuations in serum creatinine levels during hospitalization and long-term end-stage kidney disease and mortality. *JAMA Netw Open*. 2023;6(8):e2326996. doi:10.1001/jamanetworkopen.2023.26996

eTable 1. Definitions of Variables Used for the Exposure, Eligibility, and Adjustment

eTable 2. Treatment Frequency of Diuretics or Renin-Angiotensin-Aldosterone System Inhibitors on Admission and Discharge

eTable 3. The Association Between Kidney Function at Admission and Outcomes Following the Index Hospitalization With Adjustment to eGFR at Discharge

eTable 4. Additional Analyses of the Association Between Kidney Function at Admission and Outcomes Following the Index Hospitalization

eFigure. Proportionality of Hazards of the Main Exposure Assessed by Schoenfeld Residuals

This supplementary material has been provided by the authors to give readers additional information about their work.

© 2023 Efros O et al. *JAMA Network Open.*

eTable 1. Definitions of variables used for the exposure, eligibility, and adjustment

Variable	Value	Definitions	Timing
**Chronic Kidney Disease**	ICD10[a] code N18.1-N18.6<br>ICD10[a] code N18.9<br>Free text documentation in the diagnosis description field of past dialysis or kidney transplantation	0 / 1	Date of administration
**Cancer**	ICD10[a] codes C00-C97	0 / 1	Date of administration
**Ischemic Heart Disease**	ICD10[a] code I20.0<br>ICD10[a] code I20.1<br>ICD10[a] code I20.8<br>ICD10[a] code I20.9	0 / 1	Date of administration
**Heart Failure**	ICD10[a] codes I50.1-I50.4<br>ICD10[a] code I50.8<br>ICD10[a] code I50.9	0 / 1	Date of administration
**Chronic Obstructive Pulmonary Disease**	ICD10[a] codes J41-J45	0 / 1	Date of administration
**Atrial Fibrillation**	ICD10[a] codes I48.0-I48.4<br>ICD10[a] code I48.9	0 / 1	Date of administration
**Hypertension**	ICD10[a] code I10	0 / 1	Date of administration
**Cerebrovascular Accident**	ICD10[a] code I63	0 / 1	Date of administration
**Diabetes Mellitus**	ICD10[a] code E09<br>ICD10[a] code E10<br>ICD10[a] code E11<br>ICD10[a] code E13	0 / 1	Date of administration
**Angiotensin-Converting Enzyme Inhibitors**	ATC[b] code C09A<br>ATC[b] code C09B	0 / 1	Date of administration
**Angiotensin II Receptor Blockers**	ATC[b] code C09C<br>ATC[b] code C09D	0 / 1	Date of administration

[a]ICD10 International Classification of Diseases 10th Revision
[b]ATC Anatomical Therapeutic Chemical classification system

© 2023 Efros O et al. *JAMA Network Open.*

eTable 2. Treatment frequency of diuretics or renin-angiotensin-aldosterone system inhibitors on admission and discharge

Medications	Total		Normal-to-normal		Low-to-normal	
	Admission	Discharge	Admission	Discharge	Admission	Discharge
Furosemide, n (%)	4,589 (11.3)	6,996 (17.2)	3,277 (9.5)	5,367 (15.6)	1,312 (21.1)	1,629 (26.2)
Thiazide, n (%)	1,548 (3.8)	1,624 (4)	1,203 (3.5)	1,286 (3.7)	345 (5.5)	338 (5.4)
Spironolactone, n (%)	1,720 (4.2)	2,847 (7)	1,295 (3.8)	2,344 (6.8)	425 (6.8)	503 (8.1)
ACEI[a], n (%)	8,599 (21.2)	11,628 (28.7)	6,808 (19.8)	9,679 (28.2)	1,791 (28.8)	1949 (31.3)
ARB[b], n (%)	5,561 (13.7)	5,761 (14.2)	4,445 (12.9)	4,640 (13.5)	1,116 (17.9)	1,121 (18)

[a]ACEI angiotensin-converting enzyme inhibitors
[b]ARB angiotensin II receptor blockers

© 2023 Efros O et al. *JAMA Network Open.*

eTable 3. The association between kidney function at admission and outcomes following the index hospitalization with adjustment to eGFR at discharge

Adjusted analysis of the association between the “low-to-normal” group compared to the “normal-to-normal” group: mortality in the year following the index hospitalization and end-stage kidney disease in the ten years following the index hospitalization. The analysis was performed using a Cox proportional hazards model, adjusted for age, sex, and a history of high blood pressure, ischemic heart disease, heart failure, cancer, and chronic obstructive pulmonary disease (COPD). This analysis also adjusts for discharge eGFR. When a variable was used to define a subgroup, it was not used for adjustment (e.g., the analysis within the subgroup with diabetes does not adjust for diabetes).

Population	Mortality	End-Stage Kidney Disease
Entire study cohort	1.49 (95% CI: 1.40-1.58)	2.41 (95% CI: 1.58-3.68)
Granular Exposure Groups		
Discharge eGFR 45-60 mL/min/1.73m2	1.33 (95% CI: 1.24-1.43)	2.49 (95% CI: 1.55-3.98)
Discharge eGFR 0-45 mL/min/1.73m2	1.80 (95% CI: 1.65-1.95)	2.25 (95% CI: 1.21-4.20)
Age ≥ 70	1.45 (95% CI: 1.36-1.56)	2.02 (95% CI: 0.87-4.73)
Age < 70	1.57 (95% CI: 1.38-1.79)	2.90 (95% CI: 1.79-4.70)
Males	1.54 (95% CI: 1.42-1.67)	2.22 (95% CI: 1.32-3.72)
Females	1.44 (95% CI: 1.32-1.56)	2.98 (95% CI: 1.41-6.30)
Diabetes Mellitus	1.41 (95% CI: 1.27-1.58)	2.63 (95% CI: 1.54-4.50)
Hypertension	1.53 (95% CI: 1.41-1.66)	2.61 (95% CI: 1.50-4.53)
Patients taking ACEI[a] or ARB[b]	1.57 (95% CI: 1.43-1.73)	2.45 (95% CI: 1.41-4.27)

[a]ACEI angiotensin-converting enzyme inhibitors
[b]ARB angiotensin II receptor blockers

© 2023 Efros O et al. *JAMA Network Open.*

eTable 4. Additional analyses of the association between kidney function at admission and outcomes following the index hospitalization

Adjusted analysis of the association between the “low-to-normal” group and outcomes compared to the “normal-to-normal” group: mortality in the year following the index hospitalization and end-stage kidney disease in the ten years following the index hospitalization. The analysis was performed using a Cox proportional hazards model, adjusted for age, sex, and a history of high blood pressure, ischemic heart disease, heart failure, cancer, and chronic obstructive pulmonary disease (COPD). When using age groups, the following groups were included: 18-40 years, 40-65 years, 65-80 years, and 80+ years.

Analysis/Population	Mortality	End-Stage Kidney Disease
Allowing different baseline hazards per age group	1.23 (95% CI: 1.17-1.31)	3.77 (95% CI: 2.53-5.61)
Hospital stay of 5 days or less	1.12 (95% CI: 1.04-1.21)	3.98 (95% CI: 2.42-6.54)
Hospital stay of more than 5 days	1.20 (95% CI: 1.11-1.30)	3.01 (95% CI: 1.46-6.21)
“Low-to-normal” group with an increase in eGFR of 30% or more	1.29 (95% CI: 1.21-1.38)	3.69 (95% CI: 2.34-5.81)

© 2023 Efros O et al. *JAMA Network Open.*

eFigure. Proportionality of hazards of the main exposure assessed by Schoenfeld residuals

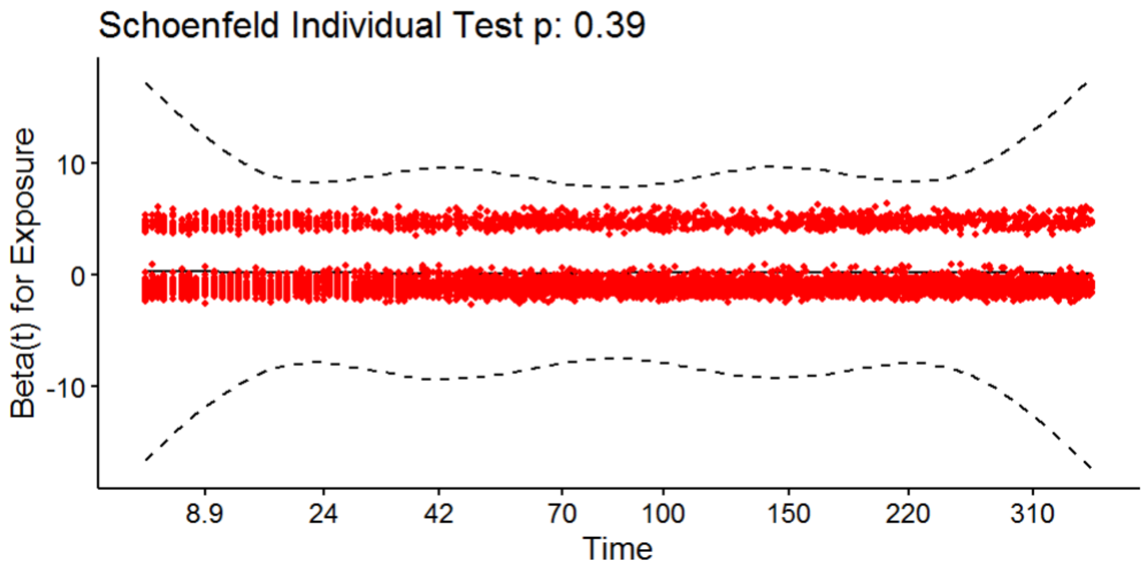

© 2023 Efros O et al. *JAMA Network Open.*
