# Supplementary material for: Fluctuations in Serum Creatinine Levels During Hospitalization and Long-Term End-Stage Kidney Disease and Mortality
Source: JAMA Netw Open. 2023 Aug 3;6(8):e2326996. doi: 10.1001/jamanetworkopen.2023.26996 (PMC10401303; doi:10.1001/jamanetworkopen.2023.26996)
Supplement: Supplement 2. — Data Sharing Statement [file jamanetwopen-e2326996-s002.pdf]

Data Sharing Statement

Efros. Fluctuations in Serum Creatinine Levels During Hospitalization and Long-Term End-Stage Kidney Disease and Mortality. *JAMA Netw Open*. Published August 03, 2023. doi:10.1001/jamanetworkopen.2023.26996

Data
Data available: Yes
Data types: Other (please specify)
Additional Information: Any requested data that will no violate the patients' confidentiality will be made available upon request.
How to access data: Request for data must be sent to the corresponding author: orly.efros@sheba.gov.il
When available: With publication

Supporting Documents
Document types: Statistical/analytic code
How to access documents: Request for data must be sent to the corresponding author: orly.efros@sheba.gov.il
When available: With publication

Additional Information
Who can access the data: Researchers whose proposed use of the data has been approved
Types of analyses: Any requested data that will no violate the patients' confidentiality will be made available upon request.
Mechanisms of data availability: With investigator support
